# The impacts of contact force, power and application time on ablation effect indicated by serial measurements of impedance drop in both conventional and high-power short-duration ablation settings of atrial fibrillation

**DOI:** 10.1007/s10840-021-00990-4

**Published:** 2021-04-23

**Authors:** Li-Bin Shi, Yu-Chuan Wang, Song-Yun Chu, Alessandro De Bortoli, Peter Schuster, Eivind Solheim, Jian Chen

**Affiliations:** 1 Department of Clinical Science, University of Bergen, Bergen, Norway; 2 Department of Heart Disease, Haukeland University Hospital, N-5021 Bergen, Norway; 3 Peking University First Hospital, Beijing, China; 4 Department of Cardiology, Oslo University Hospital, Rikshospitalet, Oslo, Norway

**Keywords:** Pulmonary vein isolation, Contact force, Power, Ablation index, High-power short-duration

## Abstract

**Background:**

This study aimed to clarify the interrelationship and additive effects of contact force (CF), power and application time in both conventional and high-power short-duration (HPSD) settings.

**Methods:**

Among 38 patients with paroxysmal atrial fibrillation who underwent first-time pulmonary vein isolation, 787 ablation points were collected at the beginning of the procedure at separate sites. Energy was applied for 60 s under power outputs of 25, 30 or 35 W (conventional group), or 10 s when using 50 W (HPSD group). An impedance drop (ID) of 10 Ω was regarded as a marker of adequate lesion formation.

**Results:**

ID ≥ 10 Ω could not be achieved with CF < 5 g under any power setting. With CF ≥ 5 g, ID could be enhanced by increasing power output or prolonging ablation time. ID for 30 and 35 W was greater than for 25 W (*p* < 0.05). Ablation with 35 W resulted in greater ID than with 30 W only when CF of 10–20 g was applied for 20–40 s (*p <* 0.05). Under the same power output, ID increased with CF level at different time points. The higher the CF, the shorter the time needed to reach ID of 10 Ω and maximal ID. ID correlated well with ablation index under each power, except for lower ID values at 25 W. ID with 50 W for 10 s was equivalent to that with 25 W for 40 s, but lower than that with 30 W for 40 s or 35 W for 30 s.

**Conclusions:**

CF of at least 5 g is required for adequate ablation effect. With CF ≥ 5g, CF, power output, and ablation time can compensate for each other. Time to reach maximal ablation effect can be shortened by increasing CF or power. The effect of HPSD ablation with 50 W for 10 s is equivalent to conventional ablation with 25 W for 40 s and 30–35 W for 20–30 s in terms of ID.

## Introduction

Radiofrequency (RF) ablation targeting ectopic atrial activities originating from the pulmonary veins (PV) has emerged as a standard approach for treating atrial fibrillation (AF) [1]. Electrical PV isolation is unanimously regarded as a cornerstone for both paroxysmal and persistent AF ablation [2, 3]. However, achieving durable PV isolation remains challenging during AF ablation, and PV electrical reconnection is frequently observed after AF recurrence, even after employing a contact-force sensing catheter [4–6].

Animal studies using both irrigated and non-irrigated ablation catheters [7–10] have shown a positive correlation between catheter-tip-tissue contact force (CF) and lesion dimensions. Although several observational studies found improvement of clinical outcomes using CF-sensing catheters, further randomized controlled studies did not confirm these initial findings [11]. Other controllable parameters, such as power and application time, also have a critical impact on ablation effectiveness. During low or standard power and long duration ablation, the power is conventionally set at 25–40 W for a duration of 20–60 s. Ablation index (AI), which integrates CF, ablation time, and power in a weighted formula, has been employed as a lesion-related indicator for guiding the ablation procedure. However, the interaction of the controllable parameters and the contribution of each to ablation efficacy have not been clearly elucidated. Recently, a new ablation strategy using high-power and short-duration (HPSD) has emerged as an option for PV isolation. The differences of efficacy between HPSD and conventional ablation settings in clinical practice though have not been demonstrated.

This study aimed to clarify the contribution of CF, power, and application duration and their interrelationship for making an adequate lesion based upon impedance drop as a surrogate for lesion formation and to compare various settings of power, contact force, and ablation duration with regard to ablation effect.

## Methods

We enrolled in this study 38 patients (24 men, mean age 65.4 ± 8.9 years) who underwent their first RF ablation procedure (PV isolation) for symptomatic paroxysmal AF. This study was approved by the Ethics Committee of Western Norway. All patients provided informed consent.

The ablation procedure performed at our institution has been previously described [12, 13]. Particular to this study, we performed a single transseptal puncture through which both ablation and circular mapping catheters were advanced into the left atrium. We carried out the procedure without the assistance of a dedicated long sheath for the ablation catheter. PV isolation was performed in all patients by ablating circumferentially at the PV antrum. To avoid mutual effects of two RF applications and the impact of the pre-existing scar issues, we identified and enrolled the ablation points in sinus rhythm at separate sites of the PVs (distance > 1 cm, local electrogram amplitude ≥ 2 mV) before circumferential ablation was performed. Ablations with visually evident displacement of the ablation catheter, stream popping, or overheating with a sudden significant impedance increase were excluded from the analysis. A 3.5-mm-tip CF-sensing irrigated ablation catheter (Navistar ThermoCool SmartTouch™, Biosense Webster, Diamond Bar, CA, USA) was employed in all the procedures. In the conventional group, RF energy was delivered in a temperature-controlled mode with a cut-off of 50 °C at a cooling rate of 2–20 mL/min. An application time of 60 s with power of 25, 30 or 35 W was used, respectively. In the HPSD group, energy was delivered in a power-controlled mode with a cooling rate of 2–30 mL/min and power of 50 W applied for 10 s.

An electroanatomic mapping system (Carto 3, Biosense Webster, Diamond Bar, CA, USA) was used and the Visitag module was activated during the procedure. Real-time CF, impedance, temperature and energy delivered were automatically updated and recorded every 20 ms and analyzed off-line. AI was calculated with a customized formula of $\mathrm{AI}={\left(k\times {\int}_0^T{CF}^{\mathrm{a}}(t){P}^{\mathrm{b}}(t) dt\right)}^{\mathrm{c}}$ by the system [14]. Impedance drop (ID) was used as the surrogate for assessment of ablation efficacy as correlation between IDs and lesion dimensions has been shown in previous studies [7, 9, 13, 15]. ID was defined as the difference between the impedance at a certain time and the baseline value. The maximum ID (MaxID) for each point represented the difference between the minimum impedance value and the impedance at baseline. Considering the variability in impedance between patients, we also calculated the maximum ID percentage (MaxID%), which was expressed by MaxID/impedance at baseline. During an application, ID ≥ 10 Ω was regarded as an adequate lesion formation [13, 16, 17].

### Statistical analysis

Continuous variables were presented as mean ± standard deviation if normally distributed; median and interquartile ranges (IQR) were used if the data were skewed according to the Shapiro–Wilk test. For comparison between groups, the analysis of variance (ANOVA) and post hoc test according to the method of Tukey’s honestly significant differences were performed. Categorical values were presented as percentages and analysed by using chi-square test or Fischer’s exact test as appropriate. The correlation among continuous variables was tested using Spearman’s rho coefficient. Statistical analysis was performed with SPSS version 24 (IBM, USA). A *p* value of < 0.05 was considered statistically significant.

## Results

A total of 787 qualified points from 38 patients (median 20 [IQR 17–22] per patient) were included in the analysis. No major complications were observed during and after the procedures. Using temperature-control mode, target power was reached after 4 s, while it took only 1 s for HPSD ablation with power-control mode. The mean CF ranged from 1.8 to 38.0 g among all applications. Four sub-groups according to mean CF value under each power setting (25, 30, 35 and 50 W) were stratified for analysis. The distribution of application points grouped for different CF level and power setting is presented in Table 1. The mean CF was 3.8 ± 0.8 vs. 3.8 ± 0.5 g in group CF < 5 g, 7.6 ± 1.4 vs. 7.0 ± 1.4 g in group CF 5–10 g, 14.2 ± 2.8 vs. 13.5 ± 2.7 g in group CF 10–20 g and 25.5 ± 4.5 vs. 25.9 ± 4.7 g in group CF ≥ 20 g (conventional vs. HPSD, *p* > 0.05). There was no difference regarding mean CF among different conventional power settings within the same CF level (*p* > 0.05).

**Table 1:** Number of applications at different levels of power and contact force and distribution of points reaching an impedance drop of 10 Ω (number and percentage in parenthesis)

		Mean contact force				
		CF < 5 g	CF 5–10 g	CF 10–20 g	CF ≥ 20 g	Total
	25 W	31 (7, 22.6%)	53 (25, 47.2%)	44 (35, 79.5%)	13 (13, 100%)	141 (80, 56.7%)
Power	30 W	16 (5, 31.3%)	55 (42, 76.4%)	55 (49, 89.1%)	14 (14, 100%)	140 (110, 78.6%)
	35 W	22 (10, 45.5%)	60 (45, 75.0%)	44 (44, 100%)	12 (12, 100%)	138 (111, 80.4%)
	50 W	16 (2, 12.5%)	157 (78, 49.7%)	157 (114, 72.6%)	38 (32, 84.2%)	368 (226, 61.4%)
Total		85 (24, 28.2%)	325 (190, 58.5%)	300 (242, 80.7%)	77 (71, 92.2%)	787 (527, 67.0%)

*CF* contact force

**Fig. 1:**
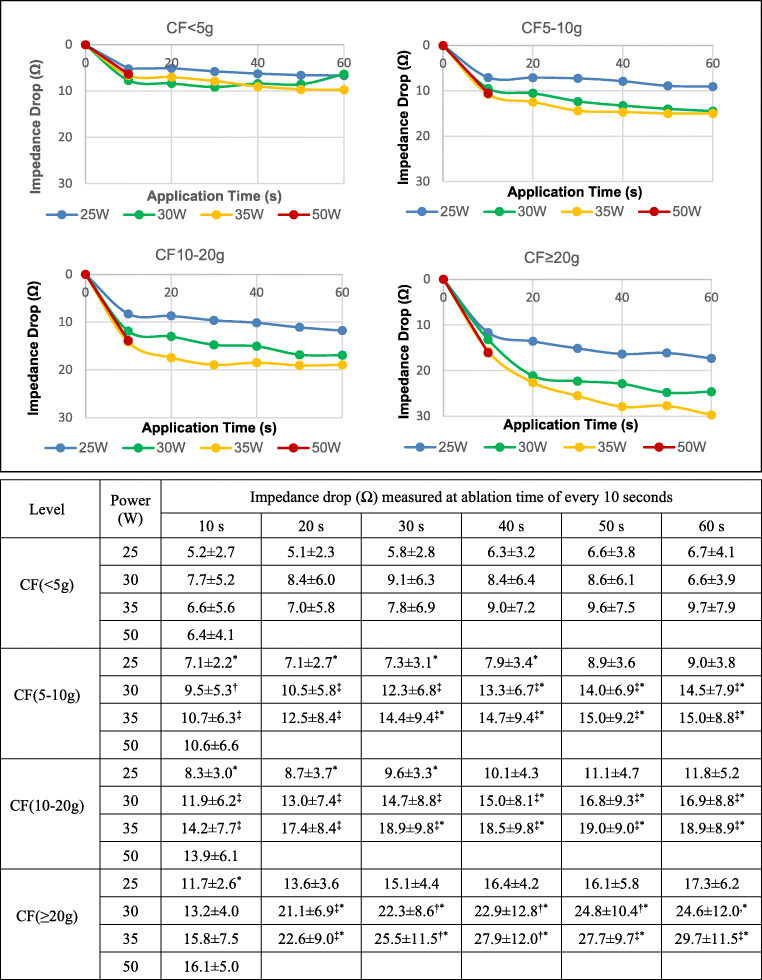
Additive impacts of contact force, power and application time on impedance drop. With CF < 5 g, ID does not reach the threshold of 10 Ω within 60 s at any power setting. When CF ≥ 5 g, power and application time compensate for each other within restricted ranges. Compared with the conventional sub-groups at different time points, ID levels at 10 s of HPSD ablation lie between those of the 25 and 30W sub-groups under the same CF level. ID, impedance drop; CF, contact force; HPSD, high power short duration; †*P* < 0.05 compared to 25 W, ‡*P* < 0.01 compared to 25 W, *P < 0.01 compared to 50W

The IDs recorded every 10 s under different power settings at different CF levels are shown in Fig. 1. We found a strong linear correlation (*ρ* = 0.978, *P* < 0.0001) between MaxID and MaxID%, which suggested that individual variability in impedance had little effect on the interpretation of our results. MaxID over 10 Ω was reached in 301 out of 419 (71.8%) ablation points in the conventional group and 226 out of 368 (61.4%) points in the HPSD group (*p* < 0.01). The proportion of ablation points in which ID reached 10 Ω is presented in Table 1. Among the conventional subgroups, a higher power and CF was observed in the points with MaxID ≥ 10 Ω compared with those < 10 Ω.

Changes of CF, power and application time individually affected ID and compensated for each other in certain circumstances.

1. Effect of prolonging application time: The effect of prolonging application time was dependent on the underlying CF and power level. With CF < 5 g, ID seldom reached 10 Ω within 60 s regardless of the power output. This was also the case for CF 5–10 g and 25 W. For CF levels beyond 5–10 g, ID increased with prolonged application under all power settings (Fig. 1). However, ablation efficacy extended marginally after 20–30 s (*p >* 0.05, compared to later time points, Fig. 1, Table 2).
2. Effect of increasing CF: It was observed for a given application time and power level (25, 30 and 35 W) that ID increased with higher CF (Fig. 1). As shown in Table 2, the time to reach ID ≥ 10 Ω and maxID tended to be shorter with increasing CF levels. A CF ≥ 20 g led to an ID ≥ 10Ω within 10 s in all power settings (Fig. 1).
3. Effect of increasing power (Fig. 1): As abovementioned, increasing power did not enlarge ID when CF < 5 g. With CF ≥ 5 g, ID under the power of 30 and 35 W were significantly higher than under 25 W (*P* < 0.01). ID under 30 and 35 W were similar (*P* > 0.05), except for CF 10–20 g for 20 to 40 s, where power of 35 W provided significantly higher ID than 30 W (*P* < 0.05).

**Table 2:** Time (in seconds) to reach impedance drop of 10 Ω and to the maximal impedance drop (in parentheses)

		Mean contact force			
		CF < 5 g	CF 5–10g	CF 10–20 g	CF ≥ 20 g
Power	25 W	―	―	40 (60)	10 (40)
	30 W	―	20 (50)	10 (50)	10 (20)
	35 W	―	10 (20)	10 (20)	10 (20)
	50 W	―	10^a^	10^a^	10^a^

*CF* contact force

^a^Total application time 10 s

The efficacy of HPSD ablation at 10 s was compared with the conventional sub-groups at different time points. ID ≥ 10 Ω was achieved at 10 s in all cases with CF ≥ 5 g in the HDSP group, but not < 5 g. With CF 5–10 g, ID in the HDSP group was higher than that under the setting of 25 W for 40 s, and lower than under 30 W for 40 s and 35 W for 30 s, respectively. With CF 10–-20 g, ID in HDSP group was higher than that under 25 W for 30 s and lower than under 30 W for 40 s and 35 W for 30 s, respectively. With CF ≥ 20 g, ID in the HDSP group was higher than that under 25 W for 10 s, and lower than under both 30 and 35 W for 20 s. Notably, differences of ID values at 10 s under powers of 30, 35 and 50 W were not statistically significant.

The average of AI in the conventional group was higher than that in the HPSD group (531.7 ± 89.8 vs. 395.8 ± 43.1, *P* < 0.01). Higher AI was found in ablation applications with ID ≥ 10 Ω than those with ID < 10 Ω in both conventional (558.7 ± 85.3 vs. 462.7 ± 58.9, *P* < 0.01) and HPSD groups (405.7 ± 43.3 vs. 380.1 ± 37.9, *P* < 0.01), respectively.

**Fig. 2:**
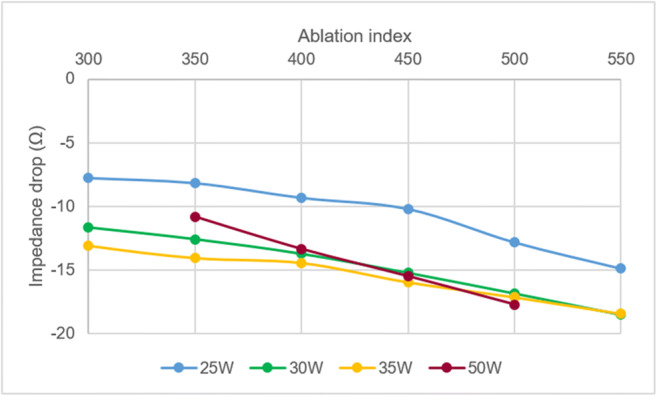
Correlation between impedance drop and ablation index under different power settings. Changes of ID with increasing AI values are similar at settings of 30, 35 and 50 W. At 25 W, they show the same trend, but with lower ID at the same AI level (*P* < 0.01)

The relationship between AI and ID under different power setting is presented in Fig. 2. The values of ID under various AI levels for powers of 30, 35 and 50 W were similar and the corresponding curves of ID were uniformly superimposed, whereas 25 W resulted in significantly lower ID at all AI levels (for AI 350–500 W/g/s, *P* < 0.01). Notably, a minimum of 450 W/g/s of AI was required to achieve ID of 10 Ω under the power of 25 W, while less than 350 W/g/s was sufficient to reach the same ID level under powers of 30–50 W.

## Discussion

In this observational study, we evaluated the additive impacts of CF, power and application time on the ablation efficacy based on ID level in patients undergoing AF ablation procedures in both conventional and HPSD settings. These three parameters compensated for each other in a CF ranging from 5 to 20 g. However, with low CF (< 5 g), ID seldom reached the threshold of 10 Ω even when power or application time was increased. The effect of HPSD ablation indicated by ID was equivalent to that of applying of 25 W for 40 s, 30 W for 30 s or 35 W for 20 s.

The essential role of CF in AF ablation has been demonstrated in a series of observational studies from basic to clinical level. However, no improvement of clinical outcomes with CF-sensing catheter was demonstrated in randomized-controlled trials [4, 18]. Experimental models have shown how ablation efficacy is dependent on several parameters [9]. During RF ablation, the surface of tissue in contact with the ablation electrode is warmed immediately by the resistant heating, while the deeper tissue layer is warmed by the conductive heating at a later stage. Passively conductive heating is time-dependent [19]. Increasing current intensity or power output at the electrode-tissue interface produces higher temperature gradients and thus greater lesion size and depth. Impedance drop is often used as a surrogate for evaluating effect of ablation as supported by earlier animal and clinical investigations [7, 9, 15, 20, 21], and previous studies have suggested an ID ≥ 10 Ω to be a reliable indicator for an adequate lesion formation [16, 17, 22]. Avitall et al. demonstrated clearly on a dog model that impedance could slightly decrease (0–10 Ω) during RF delivery even when catheter tip was 5 mm away from the tissue surface and no lesion was created. Furthermore, they found that better contact led to higher ID, and both temperature increase and ID correlated with lesion diameter and depth when ID > 10 Ω [15]. Ikeda et al. confirmed that the impedance drop during the RF application correlated well with lesion size [10]. Another clinical study conducted by Chinitz et al. showed that ID < 10 Ω accounted for 89% of sites with conduction recovery and regions with adjacent ablation with ID < 10 Ω were associated with a higher rate of conduction recovery (37% versus 1.5%) [23].

Various studies reproducibly demonstrated that a maximum lesion volume is achieved after 30 to 40 s of energy delivery and the half-time of lesion growth is around 8 s [7, 24]. In this study, the initial rapid fall of impedance was within 10 s, and the time to reach the heating plateau was in line with earlier studies. Ablation settings with power of 25, 30 and 35 W for 20 to 60 s are widely used for AF ablation. In most cases with poor CF (< 5 g), neither increasing power output nor prolonging application time enhanced the ablation effect. This result is consistent with a previous study conducted in an ex vivo model [25]. However, the study conducted by Winkle et al. showed that 14.5% impedance drop was achieved by contact force < 5 g [26]. This observational difference might be explained by several reasons. Firstly, that study used the TactiCath™ open irrigated-tip CF sensing catheter with EnSite™ Velocity™ system (St. Jude Medical). The methods of CF and impedance measurement are different from those employed in our study and the values of ID cannot be compared directly between two distinct systems. Secondly, the average application duration in that study was 12.5 s, which was longer than ours. Our study showed that application time and power had an additional effect on ID as long as CF was at least 5 g. While increase in power output resulted in consistently higher ID, the effect of increasing ablation time was insignificant after 30–40 s. This finding supports the idea of delivering RF energy to a target magnitude of AI rather than for a fixed duration. Outside this time window, the additional effect of prolonging application time on ID is limited, and indeed might lead to collateral tissue damage, especially with higher CF and power level.

Recently, more attention has been paid to the HPSD ablation strategy. Animal studies have shown the efficacy and safety of this strategy with several combinations of power (50 to 90 W) and application time (4 to 8 s) [27, 28]. Similar AF-freedom and complication rates of AF ablation using HPSD protocol with 45–50 W for 5–15 s compared with conventional strategy have been demonstrated by clinical studies [29, 30]. Bourier et al. reported that HPSD ablation, compared with standard RF application, resulted in similar lesion volumes, but with a larger maximum diameter and a smaller lesion depth. The lesion volume made by ablation using 50 W for 13 s was equal to that using 30 W for 30 s [25]. These findings were confirmed by our data which showed that ablation with 50 W for 10 s resulted in similar ID to the conventional 30 W for 30 s. However, the ID was lower than that achieved using 30 W for 40 s. Additionally, we found that a lower proportion of ablations achieved ID ≥ 10 Ω in the HPSD than in the conventional group. This may be explained by a reduction of the conductive heating phase due to the shorter application duration in the HPSD ablation with power-controlled mode [27]. Thus, for the setting of 50 W and 10 s, there might be advantage in titrated prolongation of ablation and cautious increasing of power to ensure durable lesion formation. Recently published data have shown new information supporting this hypothesis [31].

AI is a parameter integrating CF, power and application time in a weighted formula. It has been reported that predicted lesion depth based on AI correlated well with actual lesion depth in the beating canine heart. It was noteworthy that the patterns of ID responding to AI were uniform under power outputs ranging from 30 to 50 W, but not under 25 W, a finding supported by results from a recent in vivo study. This observation could be explained by the fact that the customized formula for calculating AI was based on experiments that used power outputs of 30–50 W. The improvement of clinical outcomes and durability of AI-guided PV isolation has been reported in observational studies when target values of 550 W/g/s in the anterior and 400 W/g/s in the posterior left atrial regions were employed [32, 33]. Interestingly, our data showed that the AI of 350 W/g/s might be sufficient to reach the ID goal of 10 Ω under powers of 30–50 W.

Finally, CF, power and application time contribute individually to AI by different weight. According to our results, adequate CF is an essential prerequisite. Below the CF threshold of 5 g, no significant enhancement of ablation effect can be made by increasing power or prolonging ablation time, even if total AI value has reached the recommended target value. On the other hand, with CF ≥ 5 g, the time to reach maximal ID can be reduced by increasing either CF or power. Our results showed that ablation effect could be simply enhanced about 33–88%, 49–100% and 125–227% by increasing application time from 10 to 60 s, or power from 25 to 35 W, or mean CF from 4.8 to 25.5 g, respectively (Fig. 1). Our results suggested that optimizing CF should be the first step to enhance ablation effect, and followed by adjusting power or application time, while also considering the limit of effectiveness of time (after maxID) and the restriction of power in the locations for high risk of complications (the posterior wall and thoracic veins).

### Limitations

This investigation was a single-centre non-randomized study. It focused on the effect of each single RF application without a mutual effect. Inevitably, the results could not be simply generalized to clinical outcomes. Impedance drop is a widely used parameter to monitor ablation effect but as a surrogate is flawed by several limitations as discussed previously [34, 35]. Also, impedance is measured in clinical practice with different techniques. This may influence the interpretation of optimal ID for an adequate lesion. Few points with high CF were involved in this study as catheter stability was more challenging in such situations without the support of a steerable long sheath. The ablation effect with steerable sheath may need further investigations. Power settings were selected following clinical practice, and therefore, no further information on other power levels was available. No analyses of complications with increasing CF, power and application time were performed because of extremely low incidence under the current settings.

## Conclusions

CF of at least 5 g is required for effective ablation. With CF ≥ 5 g, CF, power and application time can compensate for each other within restricted ranges. Time to reach maximal ablation effect can be shortened by increasing CF or power output. The effect of HPSD ablation with 50 W for 10 s is equivalent to conventional ablation with 25 W for 40 s and 30–35 W for 20–30 s in terms of ID. The ID versus AI increase matches well at power outputs between 30 and 50 W, but with lower ID values at 25 W.
